# Prognostic value of the fibrinogen/albumin ratio (FAR) in patients with operable soft tissue sarcoma

**DOI:** 10.1186/s12885-018-4856-x

**Published:** 2018-10-03

**Authors:** Yao Liang, Wei Wang, Yi Que, Yuanxiang Guan, Wei Xiao, Cheng Fang, Xing Zhang, Zhiwei Zhou

**Affiliations:** 1 Sun Yat-sen University Cancer Center, State Key Laboratory of Oncology in South China Collaborative Innovation Center for Cancer Medicine, Guangzhou, China; 2 Department of Gastric Surgery, Sun Yat-sen University Cancer Center, Guangzhou, China; 3 Department of Medical Melanoma and Sarcoma, Sun Yat-sen University Cancer Center, Guangzhou, China

**Keywords:** Soft tissue sarcoma, Fibrinogen/albumin ratio, Prognostic factors, Survival

## Abstract

**Background:**

Coagulation and nutrition play important roles in cancer progression. The aim of the present study is to evaluate the prognostic value of the preoperative fibrinogen/albumin ratio (FAR) in surgical patients with soft tissue sarcoma (STS) and to compare this value with other inflammatory biomarkers. In addition, we investigated the relationship between FAR and the clinicopathological characteristics of STS patients.

**Methods:**

We included 310 STS patients in this retrospective study. Kaplan-Meier curves, univariate and multivariate Cox proportional models were used in the prognostic analyses.

**Results:**

According to the receiver operating characteristic (ROC) analysis, the optimal FAR cut-off value was 0.0726. The FAR exhibited a greater area under the curve (AUC) value (0.680) than did the NLR and PLR. An elevated FAR (≥0.0726) was significantly associated with an old age, large tumor size, deep tumor location, high tumor grade, and advanced American Joint Committee on Cancer (AJCC) stage.

Patients with an increased FAR had a shorter median survival time and a lower 5-year overall survival (OS) rate than did those with a low FAR (61.0 vs115.8 months, *P* < 0.001; 56.7% vs 82.4%, *P* < 0.001, respectively). Multivariate analysis indicated FAR (Hazard ratio (HR) 1.907, 95% confidence interval (CI) 1.161–3.132, *P* < 0.001) to be an independent prognostic factor for OS, as were tumor depth, grade and PLR.

**Conclusions:**

Preoperative FAR is associated with tumor progression and can be considered an independent factor for OS of resected STS patients.

**Electronic supplementary material:**

The online version of this article (10.1186/s12885-018-4856-x) contains supplementary material, which is available to authorized users.

## Background

Soft tissue sarcomas (STSs) are rare and aggressive neoplasms of mesenchymal origin that account for approximately 1–2% of all adults cancers [1, 2]. Despite the improvement in surgical procedures and the development of adjuvant therapy [3], the long-term survival of STS patients with high-grade tumors is still poor. More than 50% of patients with high-risk STS will develop metastases and death [4–6]. Hence, it is necessary to develop effective biomarkers to identify STS patients at high risk of tumor recurrence and death after surgical treatment.

A hypercoagulable state is correlated with malignancy and it has been showed in almost 50% of the patients with tumor [7]. Among coagulation factors, fibrinogen have been in the limelight due to an essential role in inflammation as well as the development and progression of cancer. Many articles revealed that elevated preoperative plasma fibrinogen levels are significantly correlative with treatment failure or poor outcome in various malignancies [8–10], including STS [11, 12]. Additionally, accumulating data have demonstrated that preoperative malnutrition is associated with significantly short survival in STS patients [13], with serum albumin serving as a crucial parameter for nutritional status.

Therefore, we propose a novel marker, namely, the preoperative fibrinogen/albumin ratio (FAR) in combination with coagulation and nutritional status to investigate its prognostic impact in patients with operable STS. We also assessed any links between the FAR and clinicalpathological characteristics.

## Methods

### Patients

A total of 310 STS patients who underwent radical or palliative resection in Sun Yat-sen University Cancer Center (SYSUCC), Guangzhou, China from October 1999 to August 2013 were enrolled in this study. The inclusion criteria were (1) pathological diagnosis of STS; (2) surviving at least 30 days postoperatively; and (3) without neoadjuvant therapy before serum collection.

### Clinical data collection

Laboratory data, including counts of neutrophils, platelets and lymphocytes as well as fibrinogen and albumin (Alb) levels, were collected from blood samples that had been obtained before breakfast within 1 week prior to surgery. Clinical information were obtained retrospectively from the medical record. The stage of each patient was classified according to the American Joint Committee on Cancer (AJCC) 7th Edition [14] and tumors were graded according to the French Federation of Cancer Centers Sarcoma Group (FNCLCC) grading system [15]. Tumor margins were defined as follows: R0, negative/clean margins; R1, positive/involved margins (microscopic); R2, positive/involved margins (macroscopic), and Rx, the presence of residual tumor cannot be assessed). The NLR was defined by dividing the neutrophil count by the lymphocyte count. The PLR was defined by dividing the platelet count by the lymphocyte count. The FAR was defined by dividing the fibrinogen level by the Alb level.

The authenticity of this article was validated by uploading the key raw data to the Research Data Deposit public platform (www.researchdata.org.cn) with the approval RDD number of RDDA2017000360.

### Patient follow-up

The independent follow-up program department in Sun Yat-sen University provided follow-up appointments at regular intervals. The follow-up time was calculated from the date of diagnosis to the latest follow-up date (May 01, 2017) or death. The Overall survival (OS) was defined as the time interval between the initial surgery and death from any cause or the last follow-up, while the disease free survival (DFS) was defined as the time from the initial surgery until recurrence or metastasis.

### Statistical analysis

The data are expressed as the number (%), and the differences between groups were analyzed by chi-square (χ2) test or Fisher’s exact test. The optimal cutoff point was determined by the maximum Youden index (YI) based on receiver operating characteristic (ROC) analysis, and areas under the curve (AUCs) were calculated. Survival curves were analyzed according to the Kaplan-Meier method and compared by the log-rank test. Univariate and multivariate analyses of survival were conducted using the Cox proportional hazards model with forward stepwise method. Hazard ratios (HR) estimated from the Cox analysis were reported as relative risks with corresponding 95% confidence intervals (CIs). A two-sided *P*<0.05 was considered significant. All statistical operations were performed using SPSS version 20.0 (SPSS Inc., Chicago, IL, USA.).

## Results

### Patient and tumor characteristics

The demographic variables and clinicopathological characteristics of 310 STS patients enrolled in the study are listed in Table 1. There were 174 men and 136 women at a ratio of 1.3:1, with a median age of 39 years (range: 5–78 years). At the last follow-up, 98 (31.6%) had died, and 112 (36.1%) had relapsed.

**Table 1:** Baseline characteristics of all patients

Characteristics	Cases	Percentage (%)
Sex	310	
Male	174	56.1
Female	136	43.9
Age at operation (years)		
< 50	208	67.1
≥ 50	102	32.9
Performance status		
0	237	76.5
≥ 1	73	23.5
Body mass index (kg/m^2^)		
< 18.5	42	13.5
≥ 18.5 to< 25.0	208	67.1
≥ 25.0	60	19.4
Pathological types		
Fibrosarcoma	59	19.0
liposarcoma	37	11.9
Undifferentiated pleomorphic sarcoma/MFH	78	25.2
Leiomyosarcoma	17	5.5
Synovial sarcoma	40	12.9
Rhabdomyosarcoma	19	6.1
Alveolar soft part sarcoma	9	2.9
Angiosarcoma	8	2.6
Malignant peripheral nerve sheath tumor	15	4.8
Mesenchymal chondrosarcoma	8	2.6
Others	20	6.5
Tumor size (cm)		
< 5	143	46.1
≥ 5	167	53.9
Tumor site		
Upper extremity	35	11.3
Lower extremity	82	26.5
Thoracic/trunk/abdominal wall	91	29.4
Intra-abdominal	48	15.5
Head/neck	48	15.5
Others	6	1.9
Tumor depth		
Superficial	127	41.0
Deep	183	59.0
Tumor grade		
G1	82	26.5
G2	119	38.4
G3	80	25.8
Missing	29	9.4
Surgical margin		
R0	279	95.2
R1	9	3.1
R2	3	1.0
Rx	2	0.7
AJCC stage		
IA + IB	83	26.8
IIA + IIB	141	45.5
III + IV	62	20.0
Missing	24	7.1
NLR		
< 2.51	214	69.0
≥ 2.51	96	31.0
PLR		
< 191.1	265	85.5
≥ 191.1	45	14.5
FAR		
< 0.0726	176	56.8
≥ 0.0726	124	43.2
End-point		
Alive	212	68.4
Dead	98	31.6
Recurrence		
YES	112	36.1
NO	198	63.9
Metastasis		
YES	61	19.7
NO	249	80.3
Adjuvant therapy		
None	257	82.9
Chemotherapy	14	4.5
Radiotherapy	16	5.2
Combined chemoradiotherapy	2	0.6
Unknown	21	6.8

Our results demonstrated that the median fibrinogen and Alb value in the preoperative blood sample was 2.870 g/L (range: 0.18–9.87 g/L), and 42.80 g/L (range: 26.20–57.70 g/L) respectively. Regarding the different pathological tumor subtypes, there were 78 patients (25.2%) with malignant fibrous histiocytoma (MFH), 59 (19.0%) with fibrosarcoma, 40 (12.9%) with synovial sarcoma, and 37 (11.9%) with liposarcoma. Surgical resection was performed in 293 patients, R0 were detected in 279 cases (95.2%), 9 (3.1%) cases were classified R1, and only 3 cases as R2 (1.0%). The grade classification was determined to be G1 in 82 patients (26.5%), G2 in 119 patients (38.4%), and G3 in 80 patients (25.8%). Most patients had stage II disease (141, 45.5%); 83 (26.8%) had stage I disease, and 62 (20.0%) had stage III or IV disease.

The individualized radiotherapy and chemotherapy protocols were designed after operation based on the histological types, tumor stage and patient’s desire.

### Determination of the optimal cut-off value

**Fig. 1:**
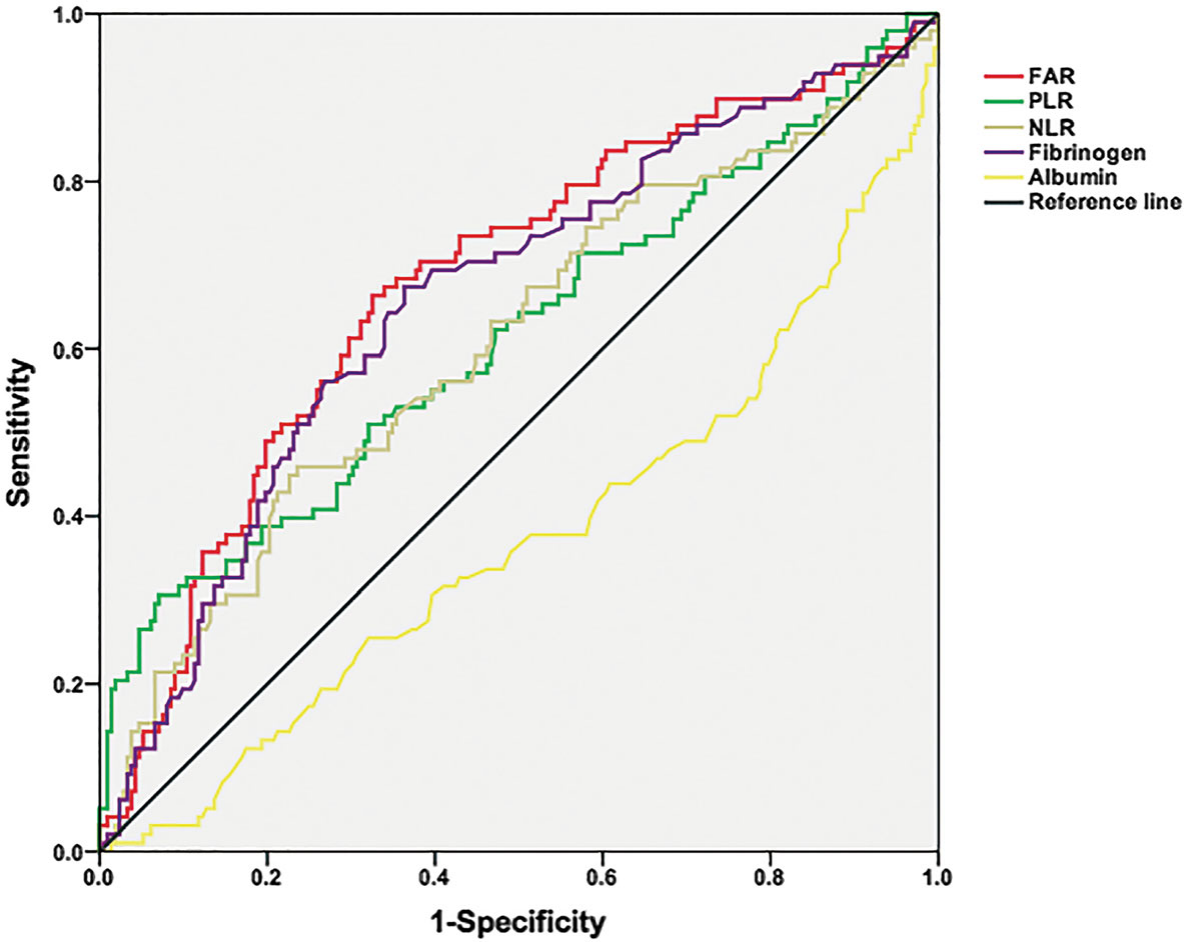
ROC curve analyses for prognostic indicators

After calculating the maximum of Youden index (sensitivity+specificity-1) and the AUC (Fig. 1 and Additional file 1: Table S1), the optimal cutoff values of the FAR, PLR and NLR were set at 0.0726 (AUC: 0.680), 191.1 (AUC: 0.614) and 2.51 (AUC: 0.608).

### Correlation between FAR and Clinicopathological characteristics

A total of 134 patients (43.2%) were categorized as high FAR (≥0.0726), and 176 patients (56.8%) were categorized as low FAR group (< 0.0726) according to the optimal cutoff value. An elevated FAR was significantly associated with age (*P* < 0.001), tumor diameter (*P* < 0.001), tumor depth (*P* < 0.001), tumor grade (*P* = 0.002), AJCC stage (*P* = 0.001) and other inflammatory markers (NLR, PLR), but not with gender (*P* = 0.779). The associations between the FAR and other variables are presented in Table 2.

**Table 2:** Correlation between the FAR and clinicopathological variables in STS patients

Variables	FAR		*p*-value
	< 0.0726, n (%)	≥0.0726, n (%)	
Gender			
Male	100 (56.8)	74 (55.2)	0.779
Female	76 (43.2)	60 (44.8)	
Age (years)			
< 50	136 (77.3)	72 (53.7)	<0.001
≥ 50	40 (22.7)	62 (46.3)	
Tumor size (cm)			<0.001
< 5	98 (55.7)	45 (33.6)	
≥ 5	78 (44.3)	89 (66.4)	
Tumor depth			<0.001
Superficial	92 (52.3)	35 (26.1)	
Deep	84 (47.7)	99 (73.9)	
Tumor grade			0.002
G1	60 (34.1)	22 (16.4)	
G2	64 (36.4)	55 (41.0)	
G3	35 (19.9)	45 (3.6)	
Missing	17 (9.7)	12 (9.0)	
AJCC stage			0.001
IA + IB	61 (34.7)	22 (16.4)	
IIA + IIB	75 (42.6)	66 (49.3)	
III + IV	25 (14.2)	37 (27.6)	
Unknown	15 (8.5)	9 (6.7)	
NLR			
< 2.51	148 (84.1)	66 (49.3)	<0.001
≥ 2.51	28 (15.9)	68 (50.7)	
PLR			<0.001
< 191.1	165 (93.8)	100 (74.6)	
≥ 191.1	11 (6.3)	34 (25.4)	
Recurrence			0.001
YES	50 (28.4)	62 (46.3)	
NO	126 (71.6)	72 (53.7)	
Metastasis			0.005
YES	25 (14.2)	36 (26.9)	
NO	151 (85.8)	98 (73.1)	

### Survival analysis

At a median follow-up period of 91.5 months, the incidence of local recurrence in the low FAR group (62 of 134, 46.3%) was significantly higher than that in the high FAR group (50 of 176, 28.4%) (*P* = 0.001).

**Fig. 2:**
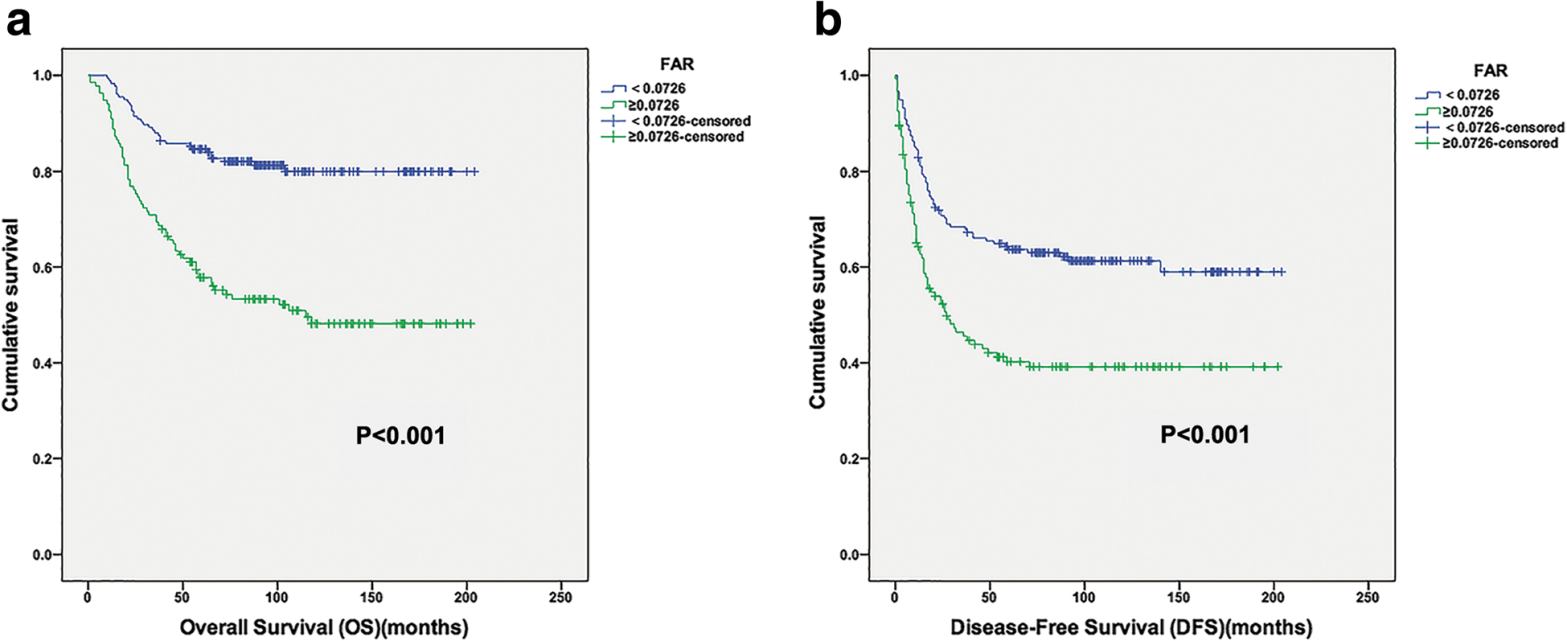
Kaplan-Meier curves showing OS (**a**) and DFS (**b**) according to the optimal value of FAR in STS patients

Regarding OS, the high-FAR group exhibited a shorter median OS and lower 5-year OS rate than the low-FAR group (61.0 vs 115.8 months, *P* < 0.001; 56.7% vs 82.4%, *P* < 0.001), respectively (Fig. 2a). Multivariate analysis revealed that preoperative FAR was an independent prognostic factor for OS (HR: 1.907; 95% CI: 1.161–3.132; *P* = 0.011). Tumor depth, tumor grade and PLR were also identified as independent predictors, whereas the tumor size, AJCC stage and NLR were not (Table 3).

**Table 3:** Univariate and multivariate analyses of variables for overall survival in STS patients

Variables	OS Univariate analysis		OS Multivariate analysis	
	HR (95% CI)	*p*-value	HR (95% CI)	*p*-value
Sex				
Male	1 (referent)			
Female	1.304 (0.877–1.937)	0.190		
Age (years)				
< 50	1 (referent)			
≥ 50	1.195 (0.793–1.02)	0.395		
Tumor size (cm)				
< 5	1 (referent)			
≥ 5	1.676 (1.109–2.535)	0.014		
Tumor depth				
Superficial	1 (referent)		1 (referent)	
Deep	3.174 (1.942–5.190)	<0.001	1.905 (1.098–3.305)	0.022
Tumor grade				
G1	1 (referent)		1 (referent)	
G2	4.339 (1.821–10.336)	0.001	3.090 (1.283–7.440)	0.012
G3	10.918 (4.647–25.652)	<0.001	6.527 (2.715–15.693)	<0.001
AJCC stage				
IA + IB	1 (referent)			
IIA + IIB	4.571 (2.065–10.115)	<0.001		
III + IV	9.540 (4.224–21.544)	<0.001		
NLR				
< 2.51	1 (referent)			
≥ 2.51	2.229 (1.497–3.318)	<0.001		
PLR				
< 191.1	1 (referent)		1 (referent)	
≥ 191.1	3.585 (2.327–5.522)	<0.001	1.936 (1.175–3.188)	0.009
FAR				
< 0.0726	1 (referent)		1 (referent)	
≥ 0.0726	3.147 (2.068–4.787)	<0.001	1.907 (1.161–3.132)	0.011

Patients in the low-FAR group demonstrated a median DFS of 76 months while a median DFS of 21 months was observed for those in the high-FAR group (Fig. 2b).

In univariate analysis, a high FAR was significantly associated with a shorter DFS (HR: 1.956; 95% CI:1.408–2.718; *P* < 0.001). However, on multivariate analysis, FAR was no longer an independent predictor of DFS, but the age, tumor depth, grade and NLR were statistically significant independent predictors of DFS (Table 4).

**Table 4:** Univariate and multivariate analyses of variables for disease-free survival in STS patients

Variables	DFS Univariate analysis		DFS Multivariate analysis	
	HR (95% CI)	*p*-value	HR (95% CI)	*p*-value
Sex				
Male	1 (referent)			
Female	1.014 (0.730–1.409)	0.934		
Age (years)				
< 50	1 (referent)		1 (referent)	
≥ 50	1.538 (1.103–2.145)	0.011	1.601 (1.111–2.306)	0.012
Tumor size (cm)				
< 5	1 (referent)			
≥ 5	2.181 (1.537–3.093)	<0.001		
Tumor depth				
Superficial	1 (referent)		1 (referent)	
Deep	3.006 (2.043–4.422)	<0.001	2.346 (1.541–3.571)	<0.001
Tumor grade				
G1	1 (referent)		1 (referent)	
G2	2.268 (1.340–3.837)	0.002	1.798 (1.053–3.069)	0.031
G3	5.300 (3.132–8.71)	<0.001	4.404 (2.565–7.561)	<0.001
AJCC stage				
IA + IB	1 (referent)			
IIA + IIB	2.294 (1.402–3.751)	0.001		
III + IV	5.089 (3.014–8.590)	<0.001		
NLR				
< 2.51	1 (referent)		1 (referent)	
≥ 2.51	1.778 (1.271–2.487)	0.001	1.719 (1.195–2.471)	0.003
PLR				
< 191.1	1 (referent)			
≥ 191.1	2.498 (1.688–3.698)	<0.001		
FAR				
< 0.0726	1 (referent)			
≥ 0.0726	1.956 (1.408–2.718)	<0.001		

**Fig. 3:**
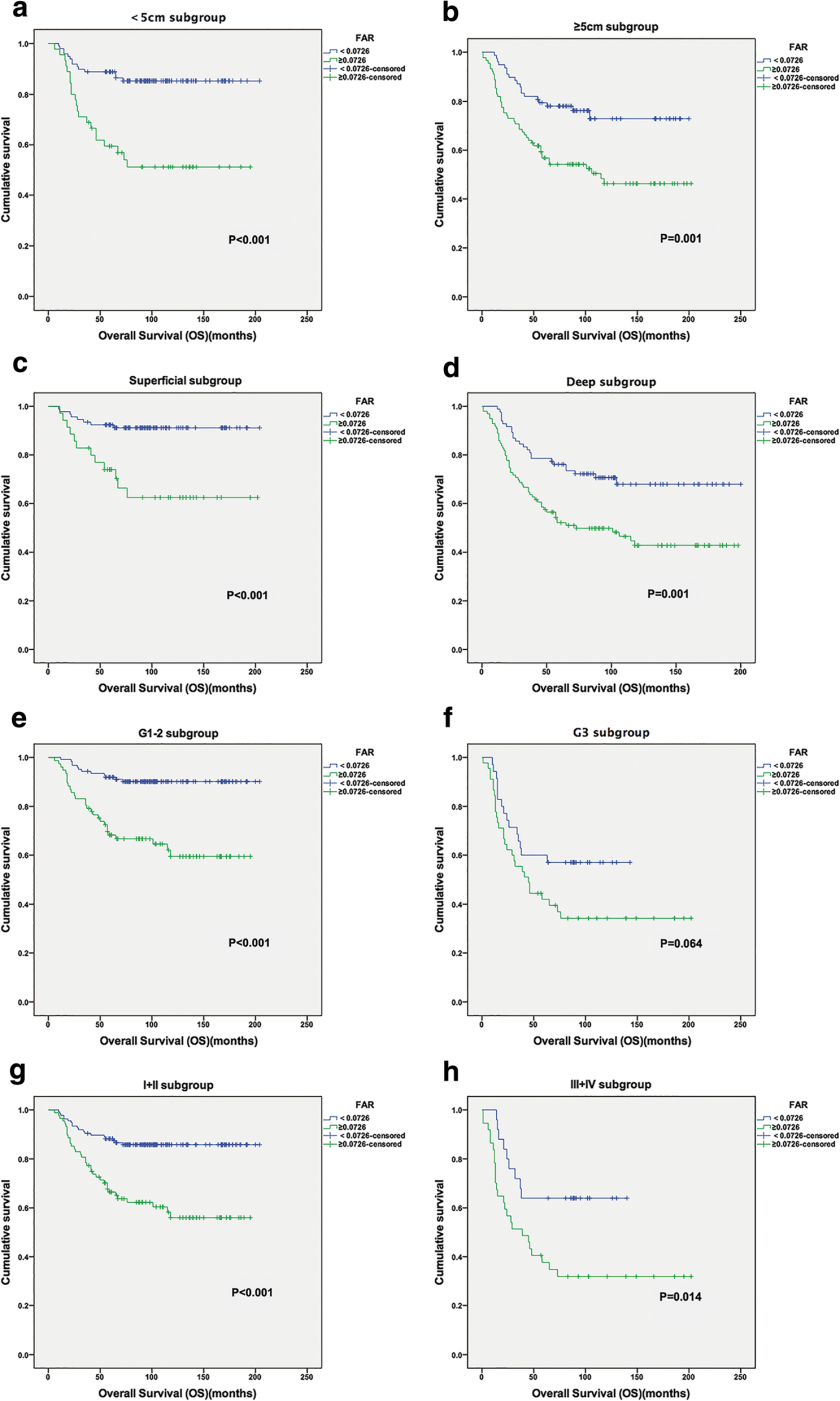
Kaplan-Meier curves showing OS according to the optimal value of FAR in < 5 cm subgroup (**a**); ≥5 cm subgroup (**b**); Superficial subgroup (**c**); Deep subgroup (**d**); G1–2 subgroup (**e**); G3 subgroup (**f**); I + II subgroup (**g**); and III + IV subgroup (**h**)

In individual subgroup analysis, we found that a longer OS was also observed in patients in the low-FAR group in the < 5 cm and ≥ 5 cm subgroups (*P* < 0.001 and *P* = 0.001), superficial and deep subgroups (*P* < 0.001 and *P* = 0.001), stage I/II and III/IV subgroups (*P* < 0.001 and *P* = 0.014), and G1/G2 subgroup (*P* < 0.001), but not in the G3 subgroup (Fig. 3).

## Discussion

To the best of our knowledge, this was the first study to evaluate the prognostic value of the FAR in STS. Our study demonstrated that preoperative FAR is independently associated with survival in resected STS patients.

Accumulating data has indicated that fibrinogen may provide favorable conditions for tumor development and progression in various malignancies [10]. However, the molecular mechanism underlying this relationship remains unclear. Fibrinogen is a dimeric molecule and serves as a scaffold for binding members of growth factors. This binding could promote tumor cell proliferation and stimulate angiogenesis [16]. Another possible mechanism may be related to the positive feedback loop between platelets and fibrinogen levels. Fibrinogen could help platelets adhere to cancer cells. In turn, these platelets induce more fibrinogen to aggregate around cancer cells, forming micro emboli in targeted organs [17]. In addition, these fibrinogen-platelet micro thrombi could functionally enhance tumor migration by preventing circulating tumor cells from natural killer cells or other cytotoxic cells [18]. A third potential mechanism that may influence prognosis is that fibrinogen may promote the inflammatory response by inducing the overproduction of pro-inflammatory cytokines in malignant cells [19]. As such, it is believed that these inflammatory responses present in the tumor microenvironment may be one of the factors responsible to provide a favorable medium for facilitating tumor proliferation, invasion, and metastases [20].

Albumin, as a chronic phase protein, is an indicator represents the nutritional status of the host, as well as a marker of systemic inflammatory response. Several studies have reported that hypoalbuminemia due to poor performance, weight loss and malnutrition is associated with poor prognosis and postoperative complications in patients with different malignancies [21–23], including STS [24]. These findings can be explained by the fact that hypoalbuminemia may weaken the human defense mechanisms, blunting the therapeutic response and further accelerating the tumor progression. Further, in patients who suffer from simultaneous nutritional decline, they are more likely to have reduced ability to endure aggressive anti-cancer therapeutics [25].

NLR and PLR are the commonly used blood sample-derived systemic inflammatory response indicators (SIR). Several studies have shown that NLR and PLR were independent prognostic factors in cancer patients [26, 27]. However, there are also some studies showing that NLR and PLR were ineffective in predicting the prognosis [28–30], indicating the prognostic role of NLR and PLR in cancer still need further investigation. They may should be assessed together with other markers due to the confounding factors regulating by inflammatory conditions.

Considering the above-mentioned evidence, in this study we successfully formulated a new technique by combining both serum fibrinogen and Alb, the FAR, which may have a potent and convincing prognostic ability. Of note, Matsuda et al. [31] recently established a prognostic indicator based on fibrinogen and Alb levels (FA score) and reported that the FA score system could predict the prognosis of patients with esophageal cancer. However, FA may estimate the coagulation and nutrition level erroneously because it evaluates the values of fibrinogen and Alb separately through categorization. Conversely, the FAR is a continuous variable that is calculated using two continuous variables, which reduces the potential bias and stratifies patients to predict survival more accurately.

According to the ROC curve, our data not only showed that the AUC value of FAR was superior to those of the NLR and PLR but also, the optimal FAR cutoff value to predict OS was at 0.0726. These results demonstrated that comparing other inflammation-based prognostic indices, the FAR had comparable prognostic capability and may be more powerful than NLR and PLR.

More importantly, our results also demonstrated that FAR might indicate a more aggressive tumor behavior and associate with the systemic progression of STS since an elevated FAR was significantly related to larger tumor size, aggressive tumor grade, more advanced AJCC stage and worsen prognoses. These findings were in agreement with previous studies demonstrating that FAR was correlated a longer tumor length, deeper tumor invasion, and increased regional lymph node involvement in ESCC patients [32].

Moreover, FAR showed a significant association with both DFS and OS in univariate analysis and was identified as an independent prognostic factor for OS in multivariate analysis. Patients with a high FAR had 1.907 times the risk of death of those with a low FAR. These results suggest that FAR has a substantial impact on patient outcome. In addition, PLR, the other index reflecting coagulation and nutrition status was also an independent prognostic factor. However, we failed to show a clear association between FAR and PFS in multivariate analysis. This may be potentially due to the small sample size, which allows a wide variability in terms of results. Another possible limitation is that unrecorded adjuvant treatments may have affected primary tumor progression.

Surprisingly, AJCC stage was not an independent predictor of OS and DFS in our analysis, which is in contrast with a previous large-scale study [33]. This could be due to the staging defects of human subjectivity and the heterogeneity of STS. The recently released 8th AJCC stage system illustrated an unprecedented change for risk stratification by redefining the T-stage categories [34]. However, the system still needs further investigation. Our data suggest that the FAR could potentially be an effective biomarker and may complement TNM staging predictions to improve risk stratification.

Further subgroup analysis found that a high FAR was associated with decreased OS in patients across all size, depth and AJCC stage subgroups. However, the FAR was not significantly associated with OS for G3 patients. These results suggest that FAR is more predictive of survival in STS patients with low-grade tumors.

It is worth noting that our findings may have important implications for individualized treatment and surveillance of patients with STS. Patients with an elevated FAR may require additional neoadjuvant therapy or more intense adjuvant chemotherapy to reduce the risk of recurrence. Both fibrinogen and Alb are routinely measured in clinical practice; the FAR has the advantage of being inexpensive, repeatable and standardized, thus offering reduced costs and increased convenience for prognostication.

This study has several limitations. First, our study used a retrospective review involving a single-center with a small sample size, which may lead to selection bias. Second, information on operation approach and adjuvant therapy was incomplete, which could lead to potential biases. Moreover, we lacked of verification analysis, which would further demonstrate our conclusions. Therefore, our results need to be verified in a large-scale prospective cohort.

## Conclusions

In summary, FAR is associated with tumor progression and can be considered an independent factor for OS of resected STS patients. Moreover, compared to the NLR and PLR, the FAR showed comparable prognostic ability based on our study population. Validation studies or large-scale prospective studies are warranted to verify our findings.

## Additional file

Additional file 1: **Table S1.** ROC analyses for prognostic indicators. (DOC 32 kb)

## Abbreviations

AJCC: American Joint Committee on Cancer
AUC: Areas under the curve
CI: Confidence interval
DFS: Disease free survival
FAR: Fibrinogen-albumin ratio
FGF: Fibroblast growth factor
HR: Hazard ratio
NLR: Neutrophil-lymphocyte ratio
OS: Overall survival
PDGF: Platelet-derived growth factor
PLR: Platelet-lymphocyte ratio
ROC: Receiver operating characteristic
STS: Soft tissue sarcoma
TGF-β: Transforming growth factor-β
TNF-α: Tumor necrosis factor-α
VEGF: Vascular endothelial growth factor

## References

[1] van der Graaf WT, Orbach D, Judson IR, Ferrari A (2017). Soft tissue sarcomas in adolescents and young adults: a comparison with their paediatric and adult counterparts. Lancet Oncol.

[2] Jo VY, Fletcher CD (2014). WHO classification of soft tissue tumours: an update based on the 2013 (4th) edition. Pathology.

[3] Chinese Journal of C, editor. The 150 most important questions in cancer research and clinical oncology series: questions 31–39 : Edited by Chinese Journal of Cancer. Chin J Cancer. 2017;36(1):48. 10.1186/s40880-017-0215-6 PMC5455093 28571582

[4] Linch M, Miah AB, Thway K, Judson IR, Benson C (2014). Systemic treatment of soft-tissue sarcoma-gold standard and novel therapies. Nat Rev Clin Oncol.

[5] Ries LA, Young JL, Keel GE, Eisner MP, Lin YD, Horner MJ (2007). SEER Survival Monograph: Cancer Survival Among Adults: U.S. SEER Program, 1988-2001, Patient and Tumor Characteristics.

[6] Cormier JN, Pollock RE (2004). Soft tissue sarcomas. CA Cancer J Clin.

[7] Jain A, Zhang Q, Toh HC (2017). Awakening immunity against cancer: a 2017 primer for clinicians. Chin J Cancer.

[8] Sheng L, Luo M, Sun X, Lin N, Mao W, Su D (2013). Serum fibrinogen is an independent prognostic factor in operable nonsmall cell lung cancer. Int J Cancer.

[9] Pichler M, Hutterer GC, Stojakovic T, Mannweiler S, Pummer K, Zigeuner R (2013). High plasma fibrinogen level represents an independent negative prognostic factor regarding cancer-specific, metastasis-free, as well as overall survival in a European cohort of non-metastatic renal cell carcinoma patients. Br J Cancer.

[10] Perisanidis C, Psyrri A, Cohen EE, Engelmann J, Heinze G, Perisanidis B, Stift A, Filipits M, Kornek G, Nkenke E (2015). Prognostic role of pretreatment plasma fibrinogen in patients with solid tumors: a systematic review and meta-analysis. Cancer Treat Rev.

[11] Asanuma K, Matsumine A, Nakamura T, Matsubara T, Asanuma Y, Oi T, Goto M, Okuno K, Kakimoto T, Yada Y (2016). Impact of plasma fibrinogen levels in benign and malignant soft tissue tumors. Cancer Biomark.

[12] Szkandera J, Pichler M, Liegl-Atzwanger B, Absenger G, Stotz M, Ploner F, Stojakovic T, Samonigg H, Eberhard K, Leithner A (2014). The elevated pre-operative plasma fibrinogen level is an independent negative prognostic factor for cancer-specific, disease-free and overall survival in soft-tissue sarcoma patients. J Surg Oncol.

[13] Szkandera J, Gerger A, Liegl-Atzwanger B, Stotz M, Samonigg H, Ploner F, Stojakovic T, Leithner A, Pichler M (2014). Pre-treatment anemia is a poor prognostic factor in soft tissue sarcoma patients. PLoS One.

[14] Edge SB, Compton CC (2010). The American joint committee on Cancer: the 7th edition of the AJCC cancer staging manual and the future of TNM. Ann Surg Oncol.

[15] Neuville A, Chibon F, Coindre JM (2014). Grading of soft tissue sarcomas: from histological to molecular assessment. Pathology.

[16] Martino MM, Briquez PS, Ranga A, Lutolf MP, Hubbell JA (2013). Heparin-binding domain of fibrin (ogen) binds growth factors and promotes tissue repair when incorporated within a synthetic matrix. Proc Natl Acad Sci U S A.

[17] Desgrosellier JS, Cheresh DA (2010). Integrins in cancer: biological implications and therapeutic opportunities. Nat Rev Cancer.

[18] Zheng S, Shen J, Jiao Y, Liu Y, Zhang C, Wei M, Hao S, Zeng X (2009). Platelets and fibrinogen facilitate each other in protecting tumor cells from natural killer cytotoxicity. Cancer Sci.

[19] Jensen T, Kierulf P, Sandset PM, Klingenberg O, Joo GB, Godal HC, Skjonsberg OH (2007). Fibrinogen and fibrin induce synthesis of proinflammatory cytokines from isolated peripheral blood mononuclear cells. Thromb Haemost.

[20] Korniluk A, Koper O, Kemona H, Dymicka-Piekarska V (2017). From inflammation to cancer. Ir J Med Sci.

[21] Miura K, Hamanaka K, Koizumi T, Kitaguchi Y, Terada Y, Nakamura D, Kumeda H, Agatsuma H, Hyogotani A, Kawakami S (2017). Clinical significance of preoperative serum albumin level for prognosis in surgically resected patients with non-small cell lung cancer: comparative study of normal lung, emphysema, and pulmonary fibrosis. Lung Cancer.

[22] Cai W, Zhang J, Chen Y, Kong W, Huang Y, Huang J, Zhou L (2017). Association of post-treatment hypoalbuminemia and survival in Chinese patients with metastatic renal cell carcinoma. Chin J Cancer.

[23] Liu DQ, Li FF, Jia WH (2016). Cumulative scores based on plasma D-dimer and serum albumin levels predict survival in esophageal squamous cell carcinoma patients treated with transthoracic esophagectomy. Chin J Cancer.

[24] Iqbal N, Shukla NK, Deo SV, Agarwala S, Sharma DN, Sharma MC, Bakhshi S (2016). Prognostic factors affecting survival in metastatic soft tissue sarcoma: an analysis of 110 patients. Clin Transl Oncol.

[25] Pant S, Martin LK, Geyer S, Wei L, Van Loon K, Sommovilla N, Zalupski M, Iyer R, Fogelman D, Ko AH (2014). Baseline serum albumin is a predictive biomarker for patients with advanced pancreatic cancer treated with bevacizumab: a pooled analysis of 7 prospective trials of gemcitabine-based therapy with or without bevacizumab. Cancer.

[26] Liang Y, Wang W, Li J, Guan Y, Que Y, Xiao W, Zhang X, Zhou Z (2018). Combined use of the neutrophil-lymphocyte and platelet-lymphocyte ratios as a prognostic predictor in patients with operable soft tissue sarcoma. J Cancer.

[27] Zhu Y, Zhou S, Liu Y, Zhai L, Sun X (2018). Prognostic value of systemic inflammatory markers in ovarian Cancer: a PRISMA-compliant meta-analysis and systematic review. BMC Cancer.

[28] Wang L, Liang D, Xu X, Jin J, Li S, Tian G, Gao Z, Liu C, He Y (2017). The prognostic value of neutrophil to lymphocyte and platelet to lymphocyte ratios for patients with lung cancer. Oncol Lett.

[29] Hirahara N, Matsubara T, Kawahara D, Nakada S, Ishibashi S, Tajima Y (2017). Prognostic significance of preoperative inflammatory response biomarkers in patients undergoing curative thoracoscopic esophagectomy for esophageal squamous cell carcinoma. Eur J Surg Oncol.

[30] Hirahara N, Matsubara T, Hayashi H, Takai K, Fujii Y, Tajima Y (2015). Impact of inflammation-based prognostic score on survival after curative thoracoscopic esophagectomy for esophageal cancer. Eur J Surg Oncol.

[31] Matsuda S, Takeuchi H, Kawakubo H, Fukuda K, Nakamura R, Takahashi T, Wada N, Saikawa Y, Omori T, Kitagawa Y (2015). Cumulative prognostic scores based on plasma fibrinogen and serum albumin levels in esophageal cancer patients treated with transthoracic esophagectomy: comparison with the Glasgow prognostic score. Ann Surg Oncol.

[32] Tan Z, Zhang M, Han Q, Wen J, Luo K, Lin P, Zhang L, Yang H, Fu J (2017). A novel blood tool of cancer prognosis in esophageal squamous cell carcinoma: the fibrinogen/albumin ratio. J Cancer.

[33] Gutierrez JC, Perez EA, Franceschi D, Moffat FL, Livingstone AS, Koniaris LG (2007). Outcomes for soft-tissue sarcoma in 8249 cases from a large state cancer registry. J Surg Res.

[34] Amin M, Edge S, Greene F (2017). AJCC Cancer staging manual.

