# Supplementary material for: Prognostic value of the fibrinogen/albumin ratio (FAR) in patients with operable soft tissue sarcoma
Source: BMC Cancer. 2018 Oct 3;18:942. doi: 10.1186/s12885-018-4856-x (PMC6169079; doi:10.1186/s12885-018-4856-x)
Supplement: Supplementary file 1 — Table S1. ROC analyses for prognostic indicators. (DOC 32 kb) [file 12885_2018_4856_MOESM1_ESM.doc]

Additional file 1: **Table S1** ROC analyses for prognostic indicators

|  | Cut-off value | AUC | 95%CI | *p*-value |
| --- | --- | --- | --- | --- |
| FAR | 0.0726 | 0.680 | 0.615-0.746 | ＜0.001 |
| PLR | 191.1 | 0.614 | 0.543-0.686 | 0.001 |
| NLR | 2.51 | 0.608 | 0.537-0.678 | 0.002 |
